# Complementary Feeding: Tradition, Innovation and Pitfalls

**DOI:** 10.3390/nu16050737

**Published:** 2024-03-04

**Authors:** Maria Elena Capra, Nicola Mattia Decarolis, Delia Monopoli, Serena Rosa Laudisio, Antonella Giudice, Brigida Stanyevic, Susanna Esposito, Giacomo Biasucci

**Affiliations:** 1 Pediatrics and Neonatology Unit, Guglielmo da Saliceto Hospital, 29121 Piacenza, Italy; m.capra@ausl.pc.it (M.E.C.); g.biasucci@ausl.pc.it (G.B.); 2 Italian Society of Pediatric Nutrition (SINUPE), 20126 Milan, Italy; 3 Pediatric Clinic, Department of Medicine and Surgery, University Hospital of Parma, 43126 Parma, Italy; 4 Department of Medicine and Surgery, University of Parma, 43126 Parma, Italy

**Keywords:** complementary feeding, infants, nutrition, baby-led weaning, food allergy

## Abstract

According to WHO, “complementary feeding (CF) is the process starting when breast milk alone or infant formula alone is no longer sufficient to meet the nutritional requirements of infants, and therefore, other foods and liquids are needed, along with breast human milk or a breastmilk substitute”. CF is one of the most important “critical and sensitive periods” in human life: indeed, timing and approaches to solid foods introduction in an infant’s nutrition are of utmost importance as potential epigenetic factors from infancy to adulthood. CF is also deeply influenced by each country and single-family traditions, culture, and beliefs. The aim of our narrative review is to analyze traditional CF practices, including innovative and alternative ones that emerged in the last decades, such as baby-led weaning or plant-based weaning, and to evaluate their effects on the risk of developing non-communicable diseases. Moreover, we will discuss pitfalls and misunderstandings that pediatricians frequently have to face when dealing with complementary feeding. Health care professionals must not have prejudices against parents’ wishes or traditions about CF; rather, they should support and educate them in case of any alternative CF choice, always pursuing the infant’s adequate growth, neuro- and taste development, and the achievement of correct eating behavior as the primary goal.

## 1. Introduction

Complementary feeding (CF) is defined by the World Health Organization (WHO) as “the process starting when breast milk alone or infant formula alone is no longer sufficient to meet the nutritional requirements of infants, and therefore, other foods and liquids are needed, along with breast milk or a breastmilk substitute” [1]. CF is one of the most important “critical periods” in human life; the timing and approaches of solid foods introduction in infant’s nutrition are a critical window in which, according to Barker’s hypothesis, a positive or negative insult can exert important epigenetic effects in terms of outcome, thus programming the individual’s future life [2]. This crucial process in each infant’s life is not always approached properly. Parents, caregivers, and pediatricians have to face and combine tradition, innovation, and, sometimes, misleading beliefs when approaching CF. Healthcare professionals’ beliefs can influence CF timing and characteristics. A recent Italian survey among pediatricians, aimed at assessing healthcare professionals’ beliefs on CF, showed that an earlier complementary feeding start, higher use of predefined schedules, and higher attention to meat and salt intake were recommended by professionals more focused on infants’ nutritional needs than on their neurodevelopmental performances [3]. Overall, health care professionals’ role in guiding parents and caregivers during CF is of utmost importance. Adequate timing and characteristics of CF are fundamental for infants worldwide. In low- and middle-income countries, correct CF is closely linked to parents’ and caregivers’ educational level, and it also represents a milestone in infants’ diarrhea and disease prevention [4]. Moreover, in a review analyzing CF practices in 80 low- and middle-income countries, the authors concluded that monitoring CF indicators across the world and implementing policies and programs to reduce wealth-related inequalities are essential to achieving children’s nutritional standards [5]. In our review, we report CF general recommendations worldwide, also focusing on the more relevant issues for clinicians both from middle- and high-income countries. The aim of our narrative review is to discuss traditional CF practices, as well as innovative and alternative ones that emerged in the last decades, such as baby-led weaning or plant-based weaning, and to evaluate their epigenetic effects on the development of non-communicable diseases (NCDs). Moreover, we will discuss pitfalls and misunderstandings that pediatricians frequently have to face when dealing with complementary feeding. The MEDLINE–PubMed database was searched to collect and select publications from 1990 to 2023. The search included randomized placebo-controlled trials, controlled clinical trials, double-blind, randomized controlled studies, and systematic reviews. The following combinations of keywords were used: “complementary feeding” AND “tradition” OR “ dietary patterns” OR “baby-led weaning” OR “plant-based diet” OR “ food allergy” OR “ preterm infants” OR “non-communicable diseases” OR “ type 1 diabetes mellitus” OR “ celiac disease” OR “ food allergy”. We also performed a manual search of the reference lists of the selected studies. The search was limited to English-language journals and full papers only.

## 2. Tradition

### 2.1. Human Milk and Infant Formula

Breastfeeding is the “ordinary” and optimal nourishment for newborns and infants, to achieve optimal growth, development, and health [1]. Indeed, human breast milk (HM) contains the most balanced nutrient concentrations and confers several benefits through its wide group of bioactive compounds, including proteins/peptides, indigestible oligosaccharides, cells, hormones, mRNAs, nucleotides, minerals, vitamins, and innate immune factors [6]. When breast milk is not available or it is not sufficient to cover an infant’s nutritional needs, infant formula must be introduced. Infant formula composition is aimed at reproducing HM’s nutritional and functional effects, even if the biochemical composition of HM is unique and cannot be entirely reproduced [7]. Many compounds can be added to infant formulas to reach this goal. Prebiotics and probiotics are mainly valued for their ability to modulate the intestinal flora and to regulate stool consistency, and frequency of evacuations. Prebiotics (e.g., fructo-oligosaccharides and galacto-oligosaccharides, and more recently other human milk oligosaccharides) are used for their ability to increase the proportion of *Lactobacilli* and *Bifidobacteria* gut colonization and decrease that of *Escherichia* and *Clostridia* species, without side effects [8]. Current evidence has linked long-chain polyunsaturated fatty acids (LCPUFAs) to the improvement in neurological development in breastfed (BF) infants compared to formula-fed (FF) infants [9]. HM nutritional composition changes dynamically over time, depending on the mammary gland physiology, maternal diet, maternal health, and many other environmental factors [10,11]. It can also vary according to prematurity, and whether hindmilk or foremilk, as well as mature, and transitional milk, or colostrum, are considered [12]. Nutritional characteristics of HM and starting infant formulas are summarized in Table 1.

### 2.2. Traditional Complementary Feeding: Definition and Characteristics

Complementary feeding (CF) is defined by WHO as “the process starting when breast milk alone or infant formula alone is no longer sufficient to meet the nutritional requirements of infants, and therefore, other foods and liquids are needed, along with breast milk or a breastmilk substitute”. According to WHO, complementary foods (CFs) are “any food or liquids, whether manufactured or locally prepared, suitable as a complement to breast milk or to a breast-milk substitute, fed to infants during the complementary feeding period” [1] and should not include low-nutrient beverages and drinks such as teas, coffee, and sugary drinks such as soda (e.g., tea and coffee contain compounds that can inhibit iron intestinal absorption) [14,15]. Thus, CFs may include finger foods, spoon-fed pureed foods, spoon-fed lumpy foods, or beverages, either prepared at home or commercially produced [16]. Traditionally, it was thought that CF should be started at 6 months of age for both BF [17] and FF infants. However, the European Society of Paediatric Gastroenterology, Hepatology and Nutrition (ESPGHAN) and the American Academy of Pediatrics (AAP) both suggest that CFs may be introduced at 4–6 months, depending on the availability of safe CFs, the infant’s growth parameters, and the achievement of appropriate developmental milestones, thus widening the CF timeframe [18,19,20].

In the so-called “traditional complementary feeding”, CFs are first introduced in the form of purees, with gradual exposure to semisolid and finger foods (food that can be managed and eaten with hands), to finally acquire the dietary model of the entire family. Parents play a key role during this process, making decisions on the timing and content of the diet; indeed, they can adjust the pre-established amount, type, and consistency of foods usually given to infants [21] with the spoon. The foods, in the form of commercial or home-made baby foods, are specifically prepared for infants, and the correct proportions are recommended by pediatricians or other health professionals. As for this CF approach, the basic traditional meal consists of vegetable broth with semolina or rice/corn/tapioca flour, meat/fish purees, and grated fruit or fruit puree; the preparation of these meals is very quick and easy, and is thus usually preferred by parents.

CFs should be introduced no earlier than 4 months and no later than 7 months of age [14,18]. At around 6 months of age, infants should be ready to eat solid foods, due to the development of the appropriate renal, digestive, and oral motor skills (such as chewing and swallowing) [22]. A paper co-drafted by the Society for Preventive and Social Pediatrics (SIPPS), the Italian Federation of Pediatricians (FIMP), the Italian Society for Developmental Origins of Health and Disease (SIDOHaD), and the Italian Society of Pediatric Nutrition (SINUPE) reported the recommendations about the appropriate age and quantitative and qualitative modalities for the introduction of complementary foods into the diets of infants aged from 6 to 24 months [23]. The macronutrient-adequate intake for term infants aged 6 to 24 months is reported in Table 2.

A month-by-month weaning schedule, which can provide useful guidance to facilitate the CF process, is summarized in Table 3 (to be considered as an example for illustrative purposes, and be adapted to local traditions, attitudes, and practices, and locally available food types).

Traditionally, CFs have been often referred to as “weaning foods”. Currently, the term “complementary foods” is preferred, as the term “weaning” implies the cessation of breastfeeding, while the goal of solid foods introduction is to supplement HM or infant formula without replacing it. HM alone is generally sufficient to meet nutritional infants’ needs for the first 6 months of life; thereafter, infants require additional sources of nutrients [24]. The period from conception to the beginning of the third year of life (the so-called “first thousand days of life”) is considered a “critical window” in which the foundations for appropriate neurological development and healthy growth throughout life are laid. This period is crucial, as the correct timing and choice of CF introduction may play a positive epigenetic influence on infants’ physical and cognitive development [25].

Early introduction of CFs may be considered for infants at high risk of iron deficiency (ID) or healthy BF infants if the mother is unable to breastfeed at 4–6 months of age [25]. However, Obbagy JE et al. conducted a systematic review on this topic, concluding that the introduction of CFs at 4 months instead of 6 months of age had no significant impact on iron status in healthy term infants (TIs) who were BF, fed with iron-fortified formula, or both. Iron-containing CFs (e.g., fortified cereals, meat), on the other hand, may help prevent ID and maintain adequate iron status in the first year of life among infants at risk of low iron intake or inadequate iron stores [26]. A similar result was found by Miniello VL et al.: no significant difference was detected between children (either BF or FF) who started CF at 4–6 months of age and those who started it at 6 months of age in terms of short-term (growth, iron status) and long-term outcomes (hypertension, overweight/obesity, type 2 diabetes mellitus) [27].

The main Italian and international dietary recommendations on complementary feeding practices are summarized in Table 4.

Iron content in some CFs is shown in Table 5 [28].

Zinc may also be deficient during the CF period. Poor zinc status may affect cognitive and motor development, as well as immune functions [28]. Although its bioavailability is high, the HM zinc content is relatively low (1–3 μg/L) compared to that in infant formulas, [10]. However, HM zinc concentration is considered adequate for most healthy term BF infants up to 6 months of age and, therefore, it is not considered a critical factor in determining the timing of CF. In well-nourished populations, there are no reports of zinc deficiency in term BF infants up to 6 months of age [29]. Nevertheless, zinc supplementation from 1 to 9 months in developing countries has been shown to reduce mortality from infectious diseases among term and small-for-gestational-age infants [30], demonstrating that zinc intake in early childhood may be inadequate in some circumstances. According to the Italian Society of Human Nutrition, the appropriate daily intake of zinc for the Italian population is 3 mg/day from 6 to 12 months of age and 5 mg/day from 1 to 3 years of age.

Therefore, during the CF period, foods such as poultry, meat, fish, or eggs should be consumed daily, as they are rich in many essential nutrients, such as iron and zinc [17]. Adequate intake of these foods improves zinc status during the first year of life, particularly in BF infants who do not receive adequate zinc from other sources. Poorer evidence was found for infants who consumed zinc-fortified formulas [26].

Zinc content in some CFs is shown in Table 6 [28].

Other micronutrient deficiencies are not common in exclusively BF infants. However, if mothers’ diets are deficient or selective, their infants may have low intakes of specific vitamins and minerals [17]. Indeed, the HM concentrations of most group B vitamins, selenium, iodine, and LCPUFAs are directly influenced by dietary maternal intake [9]. Hence, exclusively BF infants born to mothers on a strict vegan diet and not receiving appropriate supplementation, or with unrecognized pernicious anemia, can present with clinical signs of vitamin B12 deficiency [29]. Vitamin D and phylloquinone are not decisive in choosing CF timing. HM is also low in phylloquinone (vitamin K) and vitamin D content, which may increase the risk of bleeding and rickets in exclusively BF infants, respectively. In any case, it is universally agreed to administer phylloquinone at pharmacological dose (0.5–1 mg i.m., once at birth) and vitamin D in the first year of life [31].

Vitamin D is a fundamental micronutrient related to early development, bone health, and the immune system [32]. HM has generally low vitamin D concentrations (from 10 to 80 UI/L in healthy lactating mothers); therefore, all newborns, infants, and toddlers must be granted adequate vitamin D levels. Most countries have elaborated health policies to prevent vitamin D deficiency in the first year of life, advocating vitamin D supplementation for the infant, the breastfeeding mother, or both [32]. According to most of the international recommendations and the global consensus on rickets prevention, all infants should receive 400 IU/day of vitamin D oral supplementation from birth to 12 months of age, regardless of feeding and nutrition types [33]. According to Italian guidelines, in the presence of risk factors for vitamin D deficiency (non-Caucasian ethnicity with dark skin pigmentation, vegan diet, chronic kidney disease, hepatic failure, cholestasis, malabsorption syndrome, chronic drug therapies such as steroids, and infants born from mothers with multiple risk factors for vitamin D deficiency) up to 1000 IU/day of vitamin D should be administered [34].

While protein concentration in each infant formula does not change, HM protein content ranges from 14 to 16 g/L at birth to 8–10 g/L at 3–4 months, and 7–8 g/L at 6 months [35], and then remains fairly stable until 12 months of age [29]. Protein intake in the first 6 months of life is up to 66–70% lower in BF infants than in FF infants. According to the “early protein hypothesis”, excessive protein intake in the first years of life results in increased concentrations of branched-chain amino acids, leading to increased insulin growth factor 1 and insulin secretion, which can lead to accelerated weight gain and fat storage, and thus early metabolic programming of adiposity [36].

### 2.3. Complementary Feeding Patterns Worldwide

CF is a universal practice involving the global population, but it is highly influenced by cultural, individual, and socioeconomic factors. Studies carried out in Ireland [36], Tanzania [37], and Bangladesh [38] have identified several factors that may influence CF practices: maternal age and educational level, caregivers’ socioeconomic status, the opinions of friends and relatives, traditional feeding practices, influence of social networks, father’s occupation, postnatal care, and lack of professional counseling. When HM alone is no longer sufficient to meet an infant’s nutritional needs, caregivers may be induced to early CF introduction, due to cultural or personal reasons [39]. As mentioned above, the timeframe from birth to the first 2 years of life is crucial for promoting development, health, and optimal growth [24]. Inadequate CF practices during this period, such as poor hygiene behaviors, a too early CF start, and inadequate CF nutritional content, have been identified as the main causes of diarrhea, increased infection rates, malnutrition, micronutrient deficiencies, growth retardation, poor cognitive development, and increased mortality among children worldwide [39]. Studies from USA [40] and Ireland [36] show that about 20% of mothers start CF below 4 months of age. Data collected in Europe show that most infants introduce CFs before 6 months of age [41]. Similar results have been collected in Indonesia and China, while in India, CF is started too late, if compared to WHO recommendations [42]. In a study conducted by Schiess S. et al. [43], data from 1678 European healthy TIs from Italy, Germany, Belgium, Spain, and Poland were evaluated between October 2002 and June 2004 to assess the timing of CFs introduction; 588 infants were BF for at least 4 months, while 1090 infants were FF. The results showed that the introduction of CFs was delayed in BF infants (median 21 weeks, interquartile range 19–24) compared with FF infants (median 19 weeks, interquartile range 17–21); 17.2% BF infants and 37.2% of FF infants received CFs at 4 completed months of age, earlier than recommended, whereas 97.7% of BF infants and 99.3% of FF infants had received CFs at 7 completed months. Multiple regression analysis revealed that low maternal age, low educational level, and maternal smoking were predictive of early introduction of CFs at 3 and 4 months. In Europe, it has also been reported that a considerable proportion of children consume cow’s milk before the age of 12 months. In a sample of 15,700 Italian children, 0.2% consumed cow’s milk before the age of 3 months, 1.5% between 5 and 6 months, 5.8% between 7 and 8 months, and 26.8% by the age of 1 year. On the other hand, in Sweden, it has been reported that 0.5% of infants received cow’s milk before 4 months, and 30.2% before 6 months of age [41]. Moreover, the first solid food offered during CF in Europe varies greatly in each country, due to different local traditions. For example, in Italy, the most frequently offered first solid foods are fruit (73.1%), cereals (63.9%), and vegetables (40.3%) [44]; in England, rice (74%) [45], while potatoes, carrots, sweet corn, and derivatives are the most common starting CFs in Sweden [46]. In China, India, and Indonesia, diets are characterized by a reduced variety of CFs, mostly of poor nutritional quality, such as rice, cereals, and noodles. On the other hand, nutrient-dense and protein-rich foods (e.g., animal foods) are poorly consumed, especially in rural areas of China and India [42]. In this context, a 2018 Cochrane meta-analysis reported that educational interventions have a significant impact on the timing of CF introduction and hygiene practices (moderate-quality evidence), but not on the duration of exclusive breastfeeding (very low-quality evidence). Overall, according to the authors, educational interventions lead to improved CF practices [39].

### 2.4. Complementary Feeding in Preterm Infants

Preterm infants (PIs) are defined as infants born before 37 weeks of gestational age (GA). PIs represent a particularly vulnerable population with specific nutritional needs, so the appropriate management of their early nutritional needs is of utmost importance [47]. However, the timing and methods of CF in PIs are extremely heterogeneous, both between and within different countries [48,49]. Traditionally, it was believed that several goals have to be met before starting CF in PIs, such as reaching 5 kg of body weight [49], 3–6 months or [50] 5–8 months of postnatal age (PA) [51], or 3 months of corrected age (CA) [52]. Surveys conducted among parents and caregivers show that the first solid food offered to PIs is often nutritionally inadequate (e.g., with low protein and energy content) [53], and it is usually offered before 4 months of PA, even earlier than the recommended timing for TIs [49,53,54,55]. In addition, there is wide variability in iron and vitamin supplementation patterns, and CF practices among primary care pediatricians [48]. Some studies have also shown that the degree of prematurity influences the CF; PIs born at 34–36 weeks of GA are weaned at a mean CA of 4.6 months and at a mean PA of 5.7 months [56], while those born at 22–32 weeks of GA have 9.90 higher probability of receiving CFs before 4 months of PA when compared to TIs [57]. In this population, the early introduction of CFs has been associated with a higher risk of anemia, allergy, and rapid weight gain, whereas a delayed introduction of CFs after 7–10 months of PA is likely to increase the risk of avoidant feeding disorders [52]. The Italian position paper (co-drafted by the Italian Society of Neonatology—SIN, Italian Society of Pediatrics—SIP and Italian Society of Pediatric Gastroenterology, Hepatology and Nutrition—SIGENP) recommended that CF should start for PIs between 5 and 8 months of PA, or from at least 3 months of CA [48]. In conclusion, it is not yet possible to establish a precise timing for CF initiation in these patients, as the evidence is insufficient. However, an individualized approach must be adopted, carefully analyzing the neurodevelopmental milestones reached by the infant, and his or her attitude towards CFs, using the above-reported timing as a plausible but non-mandatory guideline. Indeed, these timing criteria should correspond to the disappearance of the tongue protrusion reflex, the gradual appearance of the labial seal, the reduction in the sucking reflex in favor of lateral tongue movements, and good neck control achievement in most ex-PIs [48]. However, neurodevelopmental adequacy is not the only aspect to consider, as a difficult introduction of CFs may also be explained by possible comorbidities (e.g., bronchopulmonary dysplasia) or defensive behaviors at mealtimes [58,59,60]. Infants who have undergone neonatal surgery or who are born before 30 weeks of GA are considered at risk for oro-motor feeding problems at 12 months of CA [61]. Nutritional strategies for these patients should be reviewed and adjusted regularly by a multidisciplinary medical team, including a behavioral psychologist, speech therapist, and nutritionist [62]. Furthermore, energy requirements vary according to the degree of prematurity. It has been shown that PIs often do not meet their caloric and protein requirements from birth, and these deficits are not compensated for by the time of discharge [48]. It should also be considered that if catch-up growth has not been achieved by the time CFs are introduced, it is necessary to promote a high protein and energy intake, through an appropriate formula or specific foods [49]. However, given the specific nutritional needs of PIs, particular attention should also be paid to micronutrient intake, especially iron and vitamins. In this regard, iron and vitamin supplementation is useful to ensure adequate intake up to 6 months of age, after which iron-rich foods should be provided to ensure sufficient iron intake [48]. As a 2020 Cochrane review concluded that nutritional education for families may reduce the malnutrition risk in TIs [63], it is reasonable to assume that the same concept may be used for PIs. Based on the available evidence, there is no need to delay the onset of CF in PIs to prevent overweight and obesity later in life [48]. Regardless of the relative risk of allergy development, allergenic foods and gluten should be given at any time after 4 months of CA, limiting the amount of gluten during infancy [64].

National and international guidelines [18,23,48] promote a CF based on a wide variety of CFs. However, plant-based diets (e.g., vegetarian and vegan diets) are also gaining popularity [65] among parents, who often ask pediatricians to provide their children with CF based on such dietary regimes [66]. Due to the paucity of robust data supporting the feasibility and safety of these alternative CF regimens in the PI population, they should be carefully planned by a nutrition expert professional, who should recommend the consumption of foods rich in iodine, zinc, iron, calcium, and LCPUFAs, and low in fiber. Vitamin B12 (in the case of a vegan diet) and vitamin D supplementations are also recommended [67]. ESPGHAN made a statement on the type of milk that PIs should receive during CF. According to this scientific society, infants with extrauterine growth retardation (EUGR) or at high risk of long-term growth failure should be fed with fortified HM or formula milk adapted for PIs with LCPUFAs, zinc, phosphorus, calcium, and high protein content up to 40 (but preferably 52) weeks of postmenstrual age. Infants without EUGR should receive exclusively HM, standard formula enriched with LCPUFAs, or mixed feeding (in case of inadequate amounts of HM) [68]. Recommendations for CF in PIs, according to the SIN, SIP, and SIGENP position paper, are summarized in Table 7.

**Figure 1:**
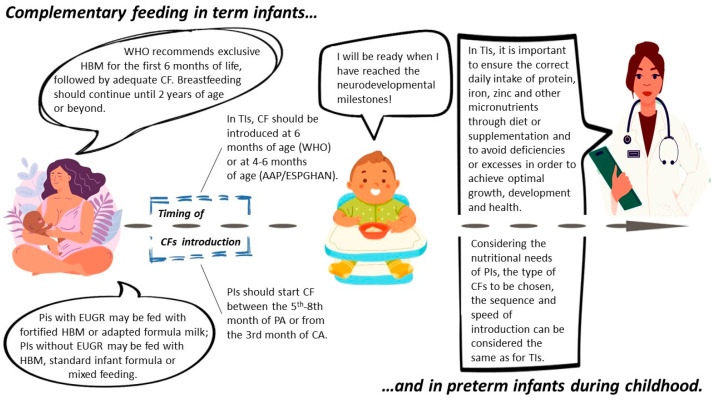
Recommendations for CF in term and preterm infants.

Recommendations for CF in term and preterm infants are summarized in Figure 1.

## 3. Baby-Led Weaning and On-Demand Complementary Feeding

CF occurs in a period of life, the so-called “first 1000 days”, that is well known as a crucial moment for children’s health and future development [69], as several epigenetic factors occurring in this timeframe may also affect later physical, cognitive, and socio-emotional health [70]. Hence, caregivers should receive adequate information about the CF process and the nutritional pattern to be adopted. Nowadays, several complementary feeding strategies are available. A correct CF approach may positively influence eating behaviors and other chronic diseases, such as overweight and obesity, allergic diseases, celiac disease, or diabetes. “Standard Weaning”, “Traditional Weaning”, “Traditional spoon feeding”, or “Parent-Led Weaning” (PLW) is widely supported by the consensus in the scientific literature. On the contrary, in the last 10–15 years, an alternative weaning approach, called “Baby-Led Weaning” (BLW) has grown up in popularity. In the UK, Rapley et al. introduced the definition of BLW as “the inclusion of the infant in family mealtimes, where food that is suitable for the infant to eat is made available to all” [71]. Through this weaning method, parents allow infants to choose where, how much, and what foods they eat, sitting at the table with the rest of the family [72]. The infant is offered the same foods as the family but as finger foods, large enough for them to be picked up by hand.

Baby-Led Introduction to SolidS (BLISS) is a modified form of BLW where caregivers are informed about choking, iron status, and failure to thrive. They are educated to cut foods into elongated formats, such as strips or sticks, and to offer children three types of food, sources of iron, energy, and fibers, at each meal [73]. BLW seems to be associated with better eating behaviors, with a lower incidence of fussiness, greater food enjoyment, and food responsiveness. In two cross-sectional studies, performed by Fu et al. and Komninou et al., infants who received BLW, followed up from 6 to 36 months, and from 12 to 36 months, respectively, had lower levels of food fussiness and major levels of food enjoyment when compared to those receiving traditional CF [74,75]. In a randomized clinical trial, Taylor et al. showed a lower satiety responsiveness in BLISS infants at 24 months (*n* = 166) compared to the spoon-weaning control group (adjusted difference, −0.24; 95% CI, −0.41 to −0.07). No body mass index (BMI) z-score differences were described between the two groups at 12 and 24 months [76]. Indeed, several studies have been carried out to investigate the growth and nutrient intake in BLW infants compared to traditionally weaned infants. An online questionnaire including sociodemographic and dietary questions has been administered to parents of 134 infants aged 6–12 months, among which 88 babies had followed Baby-Led Complementary Feeding (BLCF) and 44 babies following Standard Weaning (SW). In this cross-sectional study, the authors found no differences between the two groups in weight for age centiles, and energy, carbohydrate, protein, saturated fat, or Zn intake. However, BLCF infants received less iron from infant formulas (1.6 mg (SD 1.9) vs. 2.4 mg (SD 1.7); *p* = 0.012), and less fat and sodium from foods (*p* = 0.035 and *p* = 0.028, respectively) than SW babies [77]. In 2019, Rowan et al. conducted a study including 180 parents to compare the dietary composition of BLW to that of traditional spoon-fed children, aged 6–12 months. BLW infants were more prone to vegetables (*p* = < 0.0001) and proteins (*p* = 0.002) than traditionally weaned infants, whereas no differences were reported in exposure to iron-rich foods between the two groups [78]. In 2018, a randomized controlled trial carried out by Daniel et al. yielded the conclusion that a baby-led strategy is not correlated with the risk of iron deficiency. This trial included 206 participants assigned to control (*n* = 101) or BLISS (*n* = 105) groups [79].

Carruth and Skinner hypothesized that a baby-led approach may also permit a better development of motor ability, and both gross and fine movements, due to the repeated stimulations to use hands and fingers to handle and manage foods, as well as a concomitant better coordination of mouth and tongue movements [80].

BLW requires appropriate oral skills, including chewing and swallowing. Indeed, without proper parental control of food types and sizes, choking may become a serious risk factor for BLW. In a retrospective study, Ozyuksel et al. examined the clinical records of 75 infants aged from 5 to 12 months, who had undergone bronchoscopy due to foreign body aspiration, and they showed that 80% of aspiration occurred in BLW infants, compared to 14% during traditional CF [81]. Nevertheless, in a randomized controlled trial, Fangupo et al. showed no significant differences regarding choking episodes between the BLISS infants’ group (in which parents were educated about the relative risks) and infants following more traditional feeding practices; in the BLISS group, gagging episodes were more likely to occur at 6 months of age (relative risk (RR), 1.56; 95% confidence interval (CI), 1.13–2.17) than at 8 months (RR, 0.60; 95% CI, 0.42–0.87) [82]. The main limitation of the majority of these studies is the high prevalence of parent-reported information, which may bias the significance of the results [83].

Another alternative weaning approach, known as “self-weaning” or “on-demand complementary feeding”, emerged in Italy at the same time that BLW first arose in the UK. The main difference between the BLW strategy and the “on-demand complementary feeding”, described by the Italian pediatrician Lucio Piermarini, consists in the modality of feeding. In the BLW approach, the exclusive use of the hands is mandatory, whereas, according to on-demand complementary feeding, the use of a spoon is advisable. Indeed, times, manners, textures, and quantity of food offered during a “self-weaning” are based on the level of psycho-neuro-motor and physical development of the child (the food can be minced and mashed) [84]. This modality emphasizes the infant’s active behavior with food offers modulated as a parental response to the infant’s signs of request. Currently, there are no data available comparing BLW and on-demand CF efficacy [85].

WHO and AAP have also proposed Responsive Complementary Feeding (RCF), in which caregivers offer food only when the child is hungry and stop when the child stops demanding it. On the other hand, non-Responsive Complementary Feeding (NRCF) is characterized by a non-reciprocal relationship between caregiver and child, at least as far as mealtime is concerned. The caregiver is not involved in the child’s request or refusal of food and can force, insist, limit, or not limit food intake, or use food as a reward strategy [86].

However, should a family choose a non-traditional CF, the pediatrician’s role becomes of utmost importance, with the task of remembering the fundamental role of a healthy diet for all family members, to avoid the failure to thrive and micronutrient deficiency, and to provide proper advice to minimize choking and gabbing risks. Non-traditional CF characteristics and health implications are summarized in Table 8.

## 4. Plant-Based Complementary Feeding

Over the last decades, the prevalence of people following vegan or vegetarian diets has dramatically increased [87]. According to the results of a survey conducted by Eurispes in 2023, people following a vegetarian or vegan diet are estimated to be 6.6% of the total population in Italy (4.2% vegetarian and 2.4% vegan) [88]. There are several types of plant-based diets, all characterized by an increased intake of plant foods and a reduced or absent intake of animal products: vegan diet (no animal products are permitted), lacto-ovo-vegetarian diet (only eggs and dairy products are allowed), and ovo-vegetarian or lacto-vegetarian diet (which excludes milk or eggs, respectively). Furthermore, some people follow a primary plant diet (similar to lacto-ovo-vegetarian but with small amounts of lean meat) or pescatarian (which excludes meat and poultry, while fish is permitted). There are also some very restrictive subtypes, such as raw vegan diet (all cooked foods and processed foods are excluded), fruitarian diet (only fruits, nuts, and seeds are permitted), and macrobiotic diet (based on Taoist “yin and yang” principles, which emphasize whole grains, beans, and vegetables) [65,89]. The Italian Position Paper on Vegetarian Diets in Pregnancy and Developmental Age states that there is yet not enough scientific evidence to determine at what age it is safe to start a vegetarian diet. On the other hand, there is strong evidence that excluding certain food groups can lead to nutritional deficiencies, which may require supplementation. The German Nutrition Society also recommends that people on a vegetarian diet supplement their diet and receive regular medical checkups. However, other organizations, such as the Academy of Nutrition and Dietetics, the Portuguese National Program for the Promotion of a Healthy Diet, the Canadian Pediatric Society, and the Australian National Health and Medical Research Council, state that a well-balanced vegetarian diet is appropriate for people of all ages, including infancy [90,91,92,93,94,95]. The joint position paper of the Italian Society of Preventive and Social Pediatrics (SIPPS) with the Italian Federation of Pediatricians (FIMP) and the Italian Society of Perinatal Medicine (SIMP) has also examined the appropriateness of a vegetarian diet during childhood. The authors conclude that a vegan diet should not be recommended for children below two years of age because it would lead to deficiencies in several important nutrients (vitamin B12, vitamin D, iron, docosahexaenoic acid—DHA, and calcium) [95]. According to the American Dietetic Association, the weaning guidelines and timing of solid food introduction are the same for vegetarian and non-vegetarian children. Mangels et al. proposed a schedule of solid food introduction for vegetarian and vegan infants. Until 6 months of age, both breast milk and infant formula provide adequate intake of macro- and micronutrients, except vitamin B12, which must be given as a supplement. Between 4 and 6 months, the first solid food to be introduced should be iron-fortified infant cereals, which provide adequate energy and iron intake in an easily digestible form. Rice cereals should be firstly preferred as they usually are hypoallergenic. In case iron-fortified infant cereals are not introduced, iron supplementation becomes essential. Once the child has well tolerated the cereals taken, vegetables and fruit can be introduced, without particular attention to the order of introduction. Between 7 and 8 months of age, protein sources should be introduced. Good sources of protein for vegetarian children include soy yogurt, tofu, and legume puree. Soy-based cheeses should be introduced later, and tempeh and soy burgers by 11–12 months of age. Once the infant has reached an adequate chewing ability, the sources of carbohydrates can be more varied. A proposal for different food introductions in plant-based CF is summarized in Table 9.

Vitamin B12 is the only mandatory supplement in a plant-based diet, as even a varied and balanced diet does not guarantee sufficient intake of this micronutrient, due to the low vitamin B12 content and bioavailability in plant-based foods (algae, fungi). Thus, vitamin B12 supplementation in all people following a vegan diet, and especially in breastfeeding mothers and infants, is always mandatory. Infants’ vitamin B12 daily requirement ranges between 0.5 and 0.8 µg/day. Vitamin B12 deficiency is associated with neurological symptoms, anorexia, anemia, developmental delay, and palmar and plantar hyperpigmentation. Zinc is another micronutrient worth consideration, as its deficiency may be associated with increased susceptibility to infections, changes in taste, failure to thrive, and mucocutaneous alterations, such as dermatitis and alopecia. Fortified cereals are a good source of zinc. As for iodine, its daily requirement ranges from 50 to 80 µg/day in the first 12 months of life. Iodized salt is not recommended under 12 months of age. Fish and dairy products contain high levels of iodine, but 400 mL/day of breast milk or 900 mL/day of infant formula may also guarantee an adequate iodine intake [91]. DHA and eicosapentaenoic acid (EPA) have a significant role in retinal function, behavior, and brain development (both pre- and postnatal). Algae-derived DHA is a good option for vegan mothers’ supplementation, as the only very long-chain *n*-3 fatty acid precursor found in good amounts in plants is α-linolenic acid (ALA). ALA can be found in walnut, canola, soybean, linseed, echium seed oils, algae, paprika *Capsicum annuum*, and chia *Salvia hispanica* [9]. Indeed, breast milk and/or DHA-supplemented formula represents a good source of DHA in infants’ nutrition.

Vitamin D supplementation and adequate calcium intake are always recommended for all infants. In the first year of life, the calcium daily requirement is 500 mg/day, whereas the vitamin D daily intake should be at least 10 µg/day (400 IU/day). Calcium is mainly provided by dairy products, and the levels of phytates and oxalates are inversely proportional to its bioavailability. Rice- or soy-based infant formula provides adequate calcium intake. As for vitamin D supplementation in vegan infants, the only vitamin D drop formulation is sheep’s wool lanoline-derived vitamin D3, so vegans often refuse it. In this case, vitamin D2 (derived from fungi) supplementation, at a dosage of 2000 IU/day or 60,000 IU/month for 3 months, combined with adequate sun exposure, may represent a valid alternative for breastfeeding mothers [97], see Table 10.

Concerning protein intake, a well-balanced plant-based diet looks to be adequate. For formula-fed vegan infants, the use of rice-protein-based infant formulas, supplemented with lysine, tryptophan, and threonine, or soy-based infant formulas fortified with methionine, should be recommended.

In conclusion, the decision to start a vegetarian or vegan weaning should be made under close medical monitoring to avoid significant nutritional deficiencies. Vegetarian weaning under medical supervision is possible and should not be obstructed. Vegan weaning, on the other hand, should be carefully evaluated as related serious risks have been demonstrated (rickets, growth retardation, cognitive deficits), and it is not yet recommended by the main international scientific institutions. For ex-preterm infants, any form of alternative weaning should be discouraged. Infants following restrictive diets should be given the right vitamin supplementation throughout the weaning period, with periodic blood tests and clinical monitoring to document any micronutrient deficiencies.

## 5. Complementary Feeding Practices and Risk for Non-Communicable Diseases

NCDs are medical conditions associated with a long duration and slow progression of the illness. Most NCDs are the result of a combination of genetic, physiological, behavioral, and environmental factors. Multiple determinants interact to influence health and well-being throughout life [98]. Nutrition is a highly significant epigenetic factor in influencing health, either positively or negatively; nowadays, growing scientific evidence supports the statement that nutrition in early childhood may affect the risk of developing NCDs [24], although its epigenetic effect does not seem to be that strong, and further studies are needed in this field [99]. The CF timeframe is one of the so-called “susceptibility windows” where a positive or negative insult can have long-term effects on health outcomes in later childhood and even in adulthood.

### 5.1. Overweight and Obesity

Children with weight excess have an increased risk of becoming overweight and obese adults, and they may experience earlier onset of chronic diseases like hypertension, dyslipidemia, heart failure, and type 2 diabetes mellitus (T2DM). There are multiple risk factors for the development of overweight in infancy, both genetics and environmental; among environmental factors, dietary habits seem to act from the very early stages of life [100]. In this review, we focus on the possible correlation between the timing of CF and obesity risk, the role of the type of milk taken during CF, the type of weaning, and the quality of foods.

Data about the timing of CF and obesity risk are scarce. Sun et al. report that, in term infants, the introduction of solids at the age of 5–6 months decreases the risk of having a high BMI at 1 year of age, whereas infants weaned before 4 months have a higher risk of being overweight regardless of the duration of breastfeeding. Introduction of solid foods after 7 months is associated with increased BMI in infants breastfed for <4 months, but not in infants breastfed for ≥4 months. So, the authors concluded that longer duration of breastfeeding is associated with decreased risk of having above-normal BMI and CF should be introduced at 5–6 months of age [101].

Concerning the impact of the type of milk on weaning and weight gain, Jones et al. observed that spoon feeding was associated with increased infant weight only in formula-fed infants, while there was no significant weight difference in BLW infants who were breastfed or formula-fed. They also calculated gain velocity, as rapid weight gain during infancy may be a predictor of adiposity, but they did not find any significant differences between the two groups [102].

In a systematic review, Nazareth Martinón-Torres et al. analyzed the effect of BLW on the risk of obesity in late childhood, but their results were inconclusive [96]. In a Turkish randomized controlled study, Dogan et al. [103] showed that traditional spoon-fed infants gained more weight than the BLW group at 12 months of life. Similar results have been reported by Townsend and Pitchford [104].

Given that gut microbiota can have an impact on growth patterns in animal models and human studies [105,106,107,108], many studies have analyzed how CF and the composition of the gut microbiota may influence long-term body weight, body composition, and disease risk [109,110,111,112].

Tang et al. published a randomized controlled trial on infant growth and gut composition, in which almost 300 five-month-old infants were randomized to receive a meat-protein-predominant diet, dairy-protein-predominant diet, or plant-protein-predominant diet; they were compared to a reference infants group following traditional CFs. The authors reported an increased risk of overweight in the dairy group, even if the underlying mechanisms remain unclear. One possible cause is that they observed a higher serum Insulin-Like Growth Factor 1 (IGF-1) concentration in this group of infants, which is associated with a higher risk of obesity early in life [113]. Nevertheless, higher IGF-1 values were found from 6 to 12 months, but not at 12 or 24 months. They found that the meat and dairy groups had some specific differences in gut microbiota diversity and composition, but it is unclear how these observations could affect growth and risk of overweight. Therefore, although the plausibility of a direct protein effect on obesity during complementary feeding exists, evidence is still weak [114].

### 5.2. Type 1 Diabetes

As in other inflammatory disorders, it has been hypothesized that diet may also have epigenetic effects and affect immune dysregulation in type 1 diabetes mellitus (T1DM) pathogenesis. Breast milk seems to play a protective role against T1DM development. The MIDIA study investigated the association between breastfeeding duration and CF starting age, and the risk of islet autoimmunity and T1DM. The authors concluded that the duration of exclusive breastfeeding, solid food introduction timing, and breastfeeding at the time of introduction of any solid food cannot influence the risk of islet autoimmunity or type 1 diabetes. Breastfeeding, for 12 months or longer, is related to a lower risk of progression from islet autoimmunity to T1DM in genetically predisposed children [115]. One meta-analysis suggested an increased risk of T1DM in infants who had been early exposed to cow’s milk, but this result has not been further confirmed [116,117].

The Trial to Reduce T1DM in the Genetically at Risk (TRIGR) included 2159 newborn infants from 15 countries. In this study, CF with extensively hydrolyzed formula was not related to reduction in islet autoimmunity risk, if compared to conventional formula [118].

In 2003, the National Institutes of Health elaborated The Environmental Determinants of Diabetes in the Young (TEDDY), a multicenter prospective cohort study aimed at identifying environmental triggers of islet autoimmunity leading to T1DM; 8676 children with T1DM predisposing HLA-DR-DQ genotypes have been followed since birth in the USA, Finland, Germany, and Sweden, and environmental triggers, including infections, probiotics, micronutrients, and microbiome have been evaluated [118]. A possible protective role of breast milk was hypothesized. Breastfeeding duration was not associated with a lower risk of either islet or transglutaminase autoimmunity, while breastfeeding for more than 6 months of age, and exclusive breastfeeding for more than 3 months, were associated with decreased risk of obesity [119]. Conversely, accelerated weight gain may increase the risk for T1DM because of the establishment of insulin resistance and beta-cell overload, and consequent damage [49]. In the TEDDY study, the timing of solid food introduction was associated with islet autoimmunity in children with the HLA DR3/4 genotype not exposed to probiotics, even if the microbiome composition under these exposure combinations requires further studies [120]. In conclusion, most prospective cohort studies showed that early infant feeding practices, breastfeeding at gluten introduction, infant’s age at the time of gluten introduction, and type of milk cannot decrease the risk of developing T1DM [121].

The intake of soluble fibers has also been studied. Deficiency of soluble fiber intake has been suggested to dysregulate the local immune response, as soluble fibers are usually converted into short-chain fatty acids (SCFAs) by bacterial fermentation in the gut, with several anti-inflammatory properties. Moreover, fibers directly affect the gut microbiome, so a low fiber intake may lead to a status of dysbiosis, but no statistically significant associations between a high intake of soluble fiber and islet autoimmunity or T1DM have been found [122].

The associations between erythrocyte fatty acids and the risk of islet autoimmunity have been investigated in erythrocytes collected at the ages of 3, 6, and 12 months, and then annually up to 6 years of age. Higher EPA and DHA levels during infancy were associated with a lower risk of islet autoimmunity. Fatty acid status in early life may indicate the risk for islet autoimmunity, which may be preceded by increased levels of some short-chain and mono-unsaturated fatty acids [123].

### 5.3. Celiac Disease

Celiac disease (CD) is a disorder in which gluten intake, combined with genetic susceptibility, causes an autoimmune reaction affecting the gut and other organs. It is a permanent condition that affects approximately 1% to 3% of the general population almost worldwide, and the genetic predisposition is determined by the presence of HLA alleles DQ2 and/or DQ8 [124]. Identifying preventive strategies to reduce the prevalence of CD is one of the major targets of research in recent years, and many attempts and trials have been proposed with no univocal conclusions.

Increased serum antibody titers against cow’s milk proteins have been observed in subjects with CD [117] as well as in T1DM, and avoidance of cow’s milk-based formula has been tested [118].

In a randomized controlled trial [113], enrolling the same population of the above-mentioned TEDDY trial, the effect of extensively hydrolyzed formula on CD risk was assessed. The sample was composed of 230 infants with HLA predisposition to T1DM and at least one family member affected. Infants were divided into two groups, one fed a casein hydrolysate formula, and the other a conventional formula or breastmilk. Infants who later progressed to CD had casein antibody titers significantly higher than those of unaffected subjects. When diagnosed with CD, they also had IgG anti-beta lactoglobulin titers higher than those of non-affected infants. Nevertheless, they did not find evidence that extensively hydrolyzed formula would decrease the risk for CD later in life [118].

The timing of CF, in particular of gluten introduction, has been long debated. In 2008, ESPGHAN recommended avoiding both early (<4 months) and late (>7 months) gluten introduction, and to start it while continuing breastfeeding, to reduce CD risk [15]. The results of further observational studies showed no significant differences in CD risk in children exposed to gluten earlier than 4 months compared with first exposure at 4 to 6 months [124]. For this reason, in 2016, new evidence prompted ESPGHAN to revise recommendations, concluding that age and type of gluten introduction in infants do not seem to influence the absolute risk of developing CDA (celiac disease autoimmunity) or CD during childhood [124].

In conclusion, breastfeeding or not during gluten introduction does not reduce the risk for CD, and the avoidance of milk proteins is not protective. Hence, gluten should be introduced in a period between 4 and 8 months of age. In children at high genetic risk for CD, earlier introduction of gluten (around 4 months) is associated with earlier development of autoimmunity (defined as positive serology), but the cumulative incidence in later childhood is similar [125].

## 6. Complementary Feeding and Food Allergy

The risk of developing a food allergy (FA) is influenced by a combination of genetic and environmental factors. The recent and substantial rise in FA prevalence is primarily attributed to environmental factors. These factors could impact the food tolerance process, either directly or through epigenetic modifications [126,127]. Historically, the AAP discouraged the consumption of peanuts in children at an increased risk of atopy (i.e., those with ≥1 first-degree family relative with atopic diseases) before 3 years of age. AAP also advised against the consumption of cow’s milk throughout the entire first year of life, eggs during the second year of life, and fish and nuts during the third year of life [128]. Therefore, in the past, it was usually recommended to delay the introduction of foods with higher potential allergenic properties, as the immaturity of gut structure and function, coupled with its heightened permeability, was thought to provoke an elevated susceptibility to allergic sensitization [129]. Despite these strategies involving food avoidance, the prevalence of food allergies continued to increase in Western societies [130], suggesting that early allergen exposure might play a crucial role in attaining food tolerance, a process driven by antigens, as indicated by findings from animal models [18]. Considering this evidence, in 2008, the AAP revised recommendations on CF and FA, recognizing the uncertainty regarding the preventive aspects of allergen avoidance. They acknowledged that there was insufficient evidence to endorse maternal avoidance and delayed introduction of potential food allergens into infants’ diets as a primary tool for preventing food allergies [131]. Current guidelines suggest starting oral allergen exposure from the fourth month of age, although the most appropriate critical window for the introduction of CF for allergy prevention is still unknown [132]. Some data indicate that initiating CF before 3 or 4 months of age may increase the risk of developing allergic diseases during later infancy and childhood [133,134]. At that critical age, the intestinal barrier exhibits higher permeability, and the establishment of gastrointestinal colonization is not yet fully developed. These factors may contribute to the observed increase in the risk for allergies [135,136]. Therefore, several international guidelines aimed at allergy prevention recommend the introduction of solid foods, including egg and peanuts, after 4 months of age [135,136]. Several studies have demonstrated that delayed exposure to allergenic foods did not reduce the FA risk, both for infants with or without a positive family history of atopy [136,137]. A recent expert committee statement by the Section of Pediatrics of the European Academy of Allergology and Clinical Immunology is in close agreement with the revised AAP position [138]. In 2016, the Australasian Society of Clinical Immunology and Allergy (ASCIA) updated its guidelines for FA prevention. ASCIA recommended CF initiation “around 6 months, but not before 4 months of age,” irrespective of a family history of atopy, and preferably while continuing breastfeeding [139]. Similarly, the 2018 guidelines from the Asian Pacific Association of Pediatric Allergy, Respirology, and Immunology advised the introduction of solid foods, including those with allergenic potential, starting at six months of age, both for the general population and infants with a family history of atopic disorders [140]. The WHO strategy to prevent allergies is to promote exclusive breastfeeding during the first 6 months of the infant’s life, as a preventive strategy for the later development of allergies [141]. Current guidelines on the introduction of allergenic food during CF are summarized in Table 11.

Currently, there is insufficient conclusive evidence to support the hypothesis that early introduction of potentially allergenic foods may prevent the same food allergies, except for the introduction of peanuts between 4 and 11 months of age in infants at high risk of developing peanut allergies.

### Timing of Solid Food Introduction and Risk of Food Allergy

Peanuts, eggs, cow milk, and fish are potentially allergenic foods whose timing of introduction in CF is often analyzed and debated.

Du Toit et al. showed a ten-fold higher prevalence of peanut hypersensitivity among Jewish children living in the United Kingdom in comparison to their counterparts in Israel. Notably, in Israel, there is an important early-life peanut consumption in comparison to the limited peanut intake observed in the United Kingdom [142]. The Learning Early About Peanut Allergy Study (LEAP study), a landmark randomized, open-label, controlled trial conducted in “high-risk” infants aged 4 to 11 months with severe eczema or egg allergy or both, and with a skin-prick test (SPT) for peanut allergy <4 mm, was aimed at assessing peanut allergy in this children population. A cohort of children was randomly allocated to either receive 6 g of peanut protein per week, provided as peanut snacks or peanut butter, or to abstain from peanut intake until the age of 5 years. The authors showed that the incidence of allergies decreases when peanuts are introduced around 4 to 6 months of age. After five years, children allocated to the intervention group had a significantly lower prevalence of peanut allergy (documented with an oral food challenge) compared with those of the avoidance group (3.2% vs. 17.2%, *p* < 0.001), corresponding to a 14% and 80% absolute and relative risk reduction, respectively. It is worth noting that this difference was observed in both groups of children who initially tested negative at peanut SPT (1.9% vs. 13.7%, *p* < 0.001), and in those with SPT ranging from 1 to 4 mm (10.6% vs. 35.3%, *p* = 0.004) [130]. The LEAP-On follow-up trial further demonstrated a significantly lower (74%) peanut allergy prevalence in infants who introduced peanuts early when compared to those who had avoided peanuts intake, suggesting a persistent tolerance in the early introduction infants’ group, even one year after ceasing peanut consumption, and in the absence of repeated exposures [143]. Furthermore, early peanut introduction was observed to be allergen-specific and did not influence the development or resolution of other allergic conditions, such as asthma and atopy [144]. Following these data, a consensus statement signed by the LEAP trial team recommended peanut introduction into the diet of “high-risk” (as defined by the LEAP study) infants’ diet between 4 and 11 months of age [145]. Based on this evidence, in 2017, the American National Institute of Allergy and Infectious Diseases issued supplementary guidelines for peanut allergies [146]. These recommendations suggest an early peanut introduction at around 4–6 months of age for infants with severe atopic dermatitis and/or egg allergy (EA) following SPT or specific IgE (sIgE) testing for peanuts. If the SPT is ≤2 mm, or sIgE < 0.35 kUA/L, parents can introduce peanuts at home. On the contrary, if SPT falls between 3 and 7 mm or sIgE ≥ 0.35 kUA/L, a supervised oral peanut challenge in a medical setting is advised. Finally, infants with an SPT ≥ 8 mm have a notably elevated risk of peanut allergy and should be followed up by a pediatric allergologist [147].

Several studies investigating the impact of introducing eggs on allergy risk have yielded conflicting findings. This disparity may be likely related to uncontrolled variables within diverse study populations, as well as to variations in the dosage and form of eggs administered, including whether they were raw or cooked. In the *Hen’s Egg Allergy Prevention* (HEAP) study [148], 383 infants, aged 4 to 6 months, with no prior egg sensitization, were randomly assigned to receive freeze-dried white egg or a placebo, three times a week for six months. At one year of age, only 12 infants had developed IgE antibodies in response to eggs, with eight babies (5.6%) in the group receiving the active intervention, and four babies (2.6%) in the placebo group. The incidence of egg allergy was found to be 2.1% in the active group and 0.6% in the placebo group. In summary, this study did not provide evidence supporting the hypothesis that early egg consumption prevents food allergies and egg sensitization. Similar findings were obtained in the Australian Study Starting Time of Egg Protein (STEP) trial [149]. The Prevention of Egg Allergy with Tiny Amount Intake (PETIT) trial [150] was aimed at assessing the effectiveness and safety of introducing heated eggs as a preventive measure against egg allergies in 147 high-risk infants with atopic eczema. These infants did not exhibit immediate allergic reactions to eggs, and showed no delayed reactions to any type of food; they were randomly divided into two groups: one received heated egg powder (50 mg/day from 6 to 9 months, and 250 mg/day from 9 to 12 months), while the other received a placebo (squash). The primary outcome, which included egg allergy confirmation through open egg challenges at 12 months of age, could not be established in 26 out of 147 (17%) infants. The primary analysis included 60 infants (50%) in the egg group and 61 infants (50%) in the placebo group. By the time the infants reached 12 months of age, clinical hypersensitivity reactions to eggs were significantly less frequent in the group receiving heated eggs compared to the control group (8% vs. 38%). Furthermore, at the 12-month test, levels of egg white and ovalbumin (OVA) sIgE were notably higher in the placebo group, while OVA IgG4 antibody concentration exhibited a significant increase in the group receiving heated eggs. It is worth noting that, at baseline, levels of egg-white sIgE were higher in the control group than in the group receiving heated eggs. Additionally, it is essential to approach these findings with caution since an intention-to-treat (ITT) analysis was not conducted. Furthermore, when addressing infants with severe eczema, it would be advisable [151] to conduct egg-specific SPT/sIgE testing before egg introduction into their diet. In case sIgE and/or SPT indicate a positive reaction, both egg and peanut should be administered under medical supervision, due to the potential for clinical hypersensitivity reactions upon exposure, whereas peanut introduction may occur at home if infants exhibit negative peanut sIgE and/or peanut SPT ≤ 2 mm. Additionally, it is recommended to offer cooked food to encourage tolerance and reduce the risk of FPIES [152].

In conclusion, current guidelines recommend peanut introduction during the first year of life at home for most infants. However, for infants with severe eczema, egg allergies, or both, a medical assessment, including sensitivity testing for peanuts, should be conducted before introducing peanuts at 4–6 months of age [153]. It remains uncertain whether other allergenic foods, such as eggs, should also be introduced to an infant’s diet between 4 and 6 months of age.

When breastfeeding is unfeasible or inadequate, cow’s milk proteins are typically introduced in the early days or weeks of infants’ life, via a cow’s milk-based formula, as also advised by the AAP [152]. AAP [153] and ESPGHAN recommend refraining from relying solely on whole cow’s milk for infants’ nutrition before the age of 12 months, citing its low iron content and the potential for causing intestinal microhemorrhages [154].

Many observational studies have evaluated the timing of fish introduction in infants’ diet, and whether it affects their chances of getting asthma and allergies. In the Enquiring About Tolerance (EAT) study [155], the introduction of six allergenic foods (cow’s milk, wheat, sesame, white fish, peanut, and egg) between three and six months of age did not lower the risk of developing food allergies to these specific foods, when compared to their standard introduction after six months of age, as commonly practiced in the general population.

## 7. Conclusions

CF is a fundamental milestone in infants’ nutrition. It is a critical period in which a positive or negative insult can have effects on long-term outcomes in later life, such as growth, non-communicable diseases, and food allergies. Solid food introduction is also deeply rooted in each country and each family’s tradition and culture, but it is also influenced by new modes and trends. In this context, pediatricians should be competent guides for children and their families, enabling adequate growth and neurodevelopment, while respecting each family’s beliefs and traditions. Healthcare professionals must not have prejudices against parents’ wishes or traditions about CF; rather, they should support and educate them in case of any alternative CF choice, always pursuing the infant’s adequate growth, neuro- and taste development, and the achievement of correct eating behavior as the primary goal.

## Figures and Tables

**Table 1:** Energy, macronutrient, and micronutrient composition of HM, and recommended composition in cow’s milk formula (adapted from Koletzko et al. [13]).

Variable	Colostrum (1–5 Days)	Mature Milk (>14 Days)	Cows’ Milk–Based Starting Infant Formula (Min–Max)	Cows’Milk-Based Starting Infant Formula (Min–Max)
Energy	50–60 kcal/100 mL	65–70 kcal/100 mL	60–70 kcal/100 mL	60–70 kcal/100 mL
Carbohydrate	50–62 g/L	60–70 g/L	9.0–14.0 g/100 kcal	54–98 g/L
Total protein	14–16 g/L	8–10 g/L	1.8–3 g/100 kcal	10.8–21 g/L
Total fat	15–20 g/L	35–40 g/L	4.4–6.0 g/100 kcal	26–42 g/L
Iron	0.5–1.0 mg/L	0.3–0.7 mg/L	0.3–1.3 mg/100 kcal	2–9 mg/L
Calcium	250 mg/L	200–250 mg/L	50–140 mg/100 kcal	300–980 mg/L
Phosphorus	120–160 mg/L	120–140 mg/L	25–90 mg/100 kcal	150–630 mg/L
Magnesium	30–35 mg/L	30–35 mg/L	5–15 mg/100 kcal	30–105 mg/L
Sodium	300–400 mg/L	150–250 mg/L	20–60 mg/100 kcal	120–420 mg/L
Chloride	600–800 mg/L	400–450 mg/L	50–160 mg/100 kcal	300–1120 mg/L
Potassium	600–700 mg/L	400–550 mg/L	60–160 mg/100 kcal	360–1120 mg/L
Manganese	5–12 μg/L	3–4 μg/L	1–50 μg/100 kcal	6–350 ug/L
Iodine	40–50 μg/L	140–150 μg/L	10–50 μg/100 kcal	60–350 μg/L
Selenium	25–32 μg/L	10–25 μg/L	1–9 μg/100 kcal	9–63 μg/L

**Table 2:** Adequate intake of macronutrients in term infants from 6 to 36 months, expressed as percentage of total daily energy requirement, kcal/day or g/day (adapted from [23]).

	6–12 Months	12–24 Months	24–36 Months
**Proteins**	14%	14%	14%
**Carbohydrates**	45–55%	45–60%	45–60%
**Fats**	40%	35–40%	35–40%
**Fibers**	680–940 kcal/day	10 g/day	

**Table 3:** Month-by-month weaning schedule (adapted from [23]).

FOOD	AGE			
	6–9 Months	9–12 Months	12–18 Months	18–24 Months
**Cereals creams (rice, corn, and tapioca)**	25–30 g			
**Baby pasta and rice**		25–30 g		
**Bread**			5–10 g	
**Vegetable broth**	30–40 g			
**Fruits**	40 g (fresh fruit) 40 g (fruit puree)	50 g (twice a day) 40 g (fruit puree)	50 g three times a day	
**Vegetables**	20 g		30 g	
**Fish**	20 g (fresh fish), 40 g (fish puree)		25 g (fresh fish)	
**Meat**	10 g (fresh meat), 40 g (meat puree)		15–20 g (fresh meat)	
**Eggs**	¼ well cooked	½ well cooked		
**Legumes**	25 g (Fresh peas) 10 g (Dried legumes) 40 g (legumes puree)		30 g (Fresh peas) 30 g (Fresh green beans) 15–20 g (Dried legumes)	
**Extra virgin olive oil**	10 g			

**Table 4:** Dietary recommendations for term infants (TIs) (adapted from [15,17,18,19,23].

Dietary Recommendations for Term Infants (TIs) 0–6 Months		
American Academy of Pediatrics (AAP)	ESPGHAN	Italian Intersociety Document
- Exclusive breastfeeding is adequate to meet iron requirements until 4 months of age; - Exclusively or <50% breastfed (BF) infants: 1 mg/kg/d iron supplement for 4 to 6 months; - Formula-fed (FF) infants: formula should contains 4 to 12 mg/L of iron.	- Exclusive breastfeeding is adequate to meet iron requirements until 4 to 6 months of age; - FF infants: formula that contains 4 to 8 mg/L of iron.	- Iron store obtained in the prenatal period along with a small amount of iron from human breast milk (HM) are sufficient to meet iron needs for most healthy TIs; - Exclusive breastfeeding is adequate to meet iron requirements until 6 months of age; - Exclusively or >50% BF infants are not at risk of inadequate intakes; - FF infants: formula that contains 4 to 8 mg/L of iron.
**Dietary recommendations for TIs 7–12 months**		
**AAP**	**ESPGHAN**	**Italian Intersociety Document**
- The recommended dietary allowance (RDA) for iron is 11 mg/d.	- Dietary iron requirement 0.9–1.3 mg/kg/d; - Delay cow’s milk until 12 months of age, then limit it to 500 mL/d.	- Continued breastfeeding with the introduction of iron-rich complementary foods (CFs) at 6 months of age; - Delay cow’s milk until 12 months of age and possibly until 24 months of age, then limit it to 200–300 mL/d; - Routine iron supplementation is not recommended for healthy TIs.
WHO - Exclusive breastfeeding is adequate to meet iron requirements until 6 months of age; - Dietary recommendations for TIs 6–12 months: 8–10 mg/d of iron; - Dietary recommendations for TIs 12–24 months: 5–7 mg/d of iron; - Delay cow’s milk until 12 months of age.		

**Table 5:** Iron content of some foods used in complementary feeding (adapted from [28]).

Food	Iron Content (mg/100 g of Food)
Dried borlotti beans	9
Whole chicken egg	6.3
Oatmeal	5.2
Dried peas	4.5
Seabass	4.1
Pork meat	4
Horse meat	3.9
Lamb meat	3.2
Anchovies	3.2
Guinea fowl	2.8
Beef meat	2.3
Veal	2.3
Mackerel	2.1
Trout	2
Chicken Meat	0.23
Cow’s milk	0.1–0.2

**Table 6:** Zinc content in some complementary foods (CFs) (adapted from [28]).

Food	Zinc Content (mg/100 g of Food)
Grana Padano (cheese)	11
Lamb meat	5.8
Sardines	5.7
Turkey meat	5.1
Beef	5
Anchovies	4.2
Parmigiano Reggiano (Parmesan cheese)	4
Rabbit meat	3.9
Sardines	3.9
Dried cannellini beans	3.6
Pork meat	3.5
Guinea fowl	3.8
Dried chickpeas	3.2
Died borlotti beans/dried lentils	2.9
Chicken meat	2.8
Hen eggs, yolk	2.14

**Table 7:** Recommendations on CF in PIs, according to the position paper by SIN, SIP, and SIGENP (modified from [48]).

Item	Recommendation for Preterm Infants (PIs)	Evidence
Recommended time for initiation of complementary feeding	PIs should start complementary feeding (CF) between 5 and 8 months of postnatal age (PA) or from 3 months of correct age (CA), so that the neurodevelopmental milestones are attained.	Certainty of evidence: moderate; grade of recommendation: strong.
Management of PIs with comorbidities and/or oral dysfunction	Preterm infants with comorbidities or oral dysfunctions may require a multidisciplinary assessment to evaluate when and how CF should be started.	Certainty of evidence: low; grade of recommendation: weak.
Complementary Foods (CFs) recommended during CF	Recommendations for PIs regarding type of foods to choose, sequence and speed of introduction may be considered the same as for term infants, currently. Consider starting CF encompassing sources of carbohydrates, proteins and vegetable fats (extra-virgin olive oil) and paying special attention to the intake of micronutrients (e.g., iron and vitamins).	Certainty of evidence: low; grade of recommendation: weak.
Risk of developing overweight/obesity in relation to an early onset of CF	Timing of CF start in PIs is unlikely to influence the incidence of overweight and obesity in childhood and adulthood, so the onset of CF should not be delayed for this purpose.	Certainty of evidence: moderate; grade of recommendation: strong.
Risk of developing allergy in relation to an early onset of CF	The introduction of allergenic foods (e.g., eggs, fish, tomato, peanuts) may not be delayed in PIs.	Certainty of evidence: very low; grade of recommendation: weak.
Vegetarian and Vegan CF regimens in PIs	Vegetarian and vegan weaning may be carefully planned in PIs.	Certainty of evidence: very low; grade of recommendation: weak.
Recommended type of lactation during CF	Infants without Extra Uterine Growth Restriction (EUGR) may be fed with exclusive human breast milk (HM), standard infant formula enriched with long-chain polyunsaturated fatty acids (LCPUFAs), or mixed feeding (in case of inadequate amounts of HM). Infants with EUGR or at high risk of long-term growth failure may be fed with fortified HM or formula milk adapted for PIs as long as necessary to gain an optimal weight for CA.	Certainty of evidence: low; grade of recommendation: weak.

**Table 8:** Main characteristics of different CF strategies.

	Traditional Weaning (TW)	Baby-Led Weaning (BLW)	BLISS (Baby-Led Introduction to SolidS)	On-Demand Complementary Feeding
**Parents involvement**	Yes (spoon feeding)	No (use of hands)	No (use of hands), parents are instructed about relatives’ concerns	Yes (possibly use of spoon)
**Food texture**	Purees, semisolid, finger foods, solid	Finger foods	Finger foods	Based on the level of psycho-neuro-motor and physical development
**Benefits**	- Simpler food preparation	- Better eating behaviors - More high motor and oral development skills - Lower obesity	- Minor risk of choking, iron deficiency, and growth faltering	- Self-confidence - Active behavior
**Risks**	- More fussiness and lower food enjoyment	- Chocking - Iron deficiency - Growth faltering		- Low energy intake - Micronutrients deficiency

**Table 9:** Food introduction timing in plant-based CF, modified from [96].

Food	4–6 Months	6–8 Months	9–10 Months	11–12 Months
**Milk**	Human milk Soy formula	Human milk Soy formula	Human milk Soy formula	Human milk Soy formula
**Cereal and cereal-derived food**	Iron fortified infant cereal (usually rice is the first introduced)	Infant cereal, crackers, unsweetened dry cereal for breakfast	Infant cereal, crackers, toast, unsweetened dry cereal for breakfast, soft bread	Infant cereal, crackers, toast, unsweetened dry cereal for breakfast, soft bread, rice pasta
**Fruits and vegetables**	-	Strained fruit or vegetables, fruit or vegetable juice	Soft or cooked fruit, fruit juice, cooked mashed vegetable, vegetable juice	Soft, canned or cooked fruit, peeled raw fruit, fruit juice, cooked pieces of vegetable, vegetables juice
**Pulses**	-	Pureed legumes	Pureed legumes,	Mashed legumes
**Other food items with high protein content**		Tofu, soy yogurt (after 8 months)	Soy cheese, soy yogurt (after 8 months)	Tofu, soy cheese or yogurt, tempeh
**Other food items with high fat content**	Olive oil	Olive oil	Olive oil	Olive oil Small amount of light margarine

**Table 10:** Micronutrient requirements and supplementation for plant-based complementary feeding.

	Daily Requirement 0–12 Months	Plant-Based Food That Contains	Supplementation
Vitamin B12	0.5–0.8 µg/day	Algae, fungi, tempeh	Necessary
Calcium	500 mg/day	Dairy products, broccoli, kale, cabbage, soy drinks, tofu. Nuts, dried beans, spinach (low bioavailability)	Not necessary
Vitamin D	10 µg/day (400 IU/day)	Dairy products or cereals, fortified soy drink	Suggested
Iron	6–8 mg/day	Soaking pulses, iron-rich vegetables, iron-fortified food (cereals)	Not necessary
Zinc	5 mg/day	Zinc-fortified cereal, whole seeds, nuts, legumes, dairy products	Not necessary
Iodine	50–80 µg/day	Dairy products, breast milk, infant formula	Not necessary

**Table 11:** Recommendations on allergenic foods introduction in complementary feeding.

Guidelines	
American Academy of Pediatrics (AAP) 2019	Severe eczema and/or egg allergies: introduced to peanuts between 4 and 6 months of age, after they have successfully incorporated other solid foods into their diet and have reached the appropriate developmental stage. Highly advisable to conduct allergy testing before introducing peanuts to this specific group. Mild to moderate eczema: peanut introduction at approximately 6 months of age (aligning with the family’s preferences and cultural traditions) to reduce peanut allergy. No risk: introduced peanuts based on preferences and cultural traditions in association with other solid foods.
Asia Pacific Association of Pediatric Allergy, Respirology & Immunology (APAPARI); 2017	Severe eczema: supervised oral challenge for eggs and peanuts; introduced allergenic foods according to negative challenge results. Family history of atopy: it shouldn’t be delayed introduction of allergenic food. No risk: complementary food should be begun at 6 months of age.
National Institute of Allergy and Infectious Diseases (NIAID), 2017	Severe eczema/egg allergy: sIgE or SPT to peanut should be performed before introducing them. - sIgE < 0.35 kUA/L or peanut SPT wheal< 2 mm → introduce peanuts at home - sIgE > 0.35 kUA/L or peanut SPT wheal > 3 mm → supervised oral challenge. - SPT > 8 mm: suspected peanut allergy → continue to be managed by a specialist Mild to moderate eczema: Introduction of peanuts at 6 months. No eczema: introduction of peanuts according to age and preferences/habits of the family.
European Society for Paediatric Gastroenterology, Hepatology, and Nutrition (ESPGHAN), 2017	Complementary food should be introduced between 4 to 6 months Allergenic food should be introduced after 4 months. Peanut allergy is a high-risk factor for infants with an egg allergy or severe eczema→introduce peanuts between 4 and 11 months and should be managed by a specialist.
Asian Pacific Association of Pediatric Allergy, Respirology and Immunology (APAPARI), 2018	Infants without risk factors or with a family history of atopy→ complementary food should be introduced around 6 months. High-risk infants with severe eczema→sIgE or SPT to peanut should be performed and supervised oral challenge if it is necessary. It is important to introduce allergenic foods without delay. Supervised oral challenge should be managed only by a specialist in infants with peanut/egg allergies.
German Society for Allergology and Clinical Immunology (DGAKI)	Early introduction (after 4th month) of allergenic food may have a preventive effect Introduction of fish in the first years of life may ward off atopic diseases.
European Academy of Allergy and Immunology (EAACI)	A well-cooked hen’s egg, but not a raw egg or uncooked pasteurized egg, should be introduced after 4th month to prevent egg allergy. It is not to be suggested the introduction of cow’s milk during the first week of life to prevent cow’s milk allergy. Early introduction of peanuts during complementary food should be an important strategy to prevent peanut allergy among people with a high prevalence of peanut allergy.

## Abbreviations

AAP	American Academy of Pediatrics
AD	Atopic Dermatitis
ALA	Alfa-Linolenic Acid
BB	Breastfed
BLCF	Baby-Led Complementary Feeding
BLISS	Baby-Led Introduction to SolidS
BLW	Baby-Led Weaning
CD	Celiac Disease
CF	Complementary feeding
CFs	Complementary foods
CM	Cow Milk
DHA	Docosahexaenoic Acid
EA	Egg Allergy
EAT	Enquiring About Tolerance
EFSA	European Food Safety Authority
ESPGHAN	European Society for Paediatric Gastroenterology Hepatology and Nutrition
EUGR	Extra Uterine Growth Restriction
FA	Food Allergy
FIMP	Italian Federation of Pediatricians
FF	Formula-fed
HEAP	Hens Egg Allergy Prevention
HM	Human Breast Milk
ID	Iron Deficiency
IDA	Iron-Deficiency Anemia
IGF 1	Insulin-Like Growth Factor 1
LA	Linoleic Acid
LCPUFAs	Long-Chain Polyunsaturated Fatty Acids
LEAP	Learning Early About Peanut Allergy Study
NCDs	Non-Communicable Diseases
NRCF	Non-Responsive Complementary Feeding
PETIT	Prevention of Egg Allergy with Tiny Amount Intake
PLW	Parent-Led Weaning
PCPs	Primary Care Pediatricians
PIs	Preterm Infants
RCF	Responsive Complementary Feeding
SACN	UK Scientific Advisory Committee on Nutrition
SIDOAhD	Italian Society for Developmental Origins of Health and Disease
SIGENP	Italian Society of Pediatric Gastroenterology, Hepatology and Nutrition
SIMP	Italian Society of Perinatal Medicine
SIN	Italian Society of Neonatology
SIP	Italian Pediatric Society
SPT	Skin Prick Tests
STEP	Australian Study Starting Time of Egg Protein
SW	Standard Weaning
TEDDY	The Environmental Determinants of Diabetes in the Young
T1DM	Type 1 Diabetes Mellitus
T2DM	Type 2 Diabetes Mellitus
TRIGR	Trial to Reduce IDDM in the Genetically at-Risk
Tis	Term Infants
WHO	World Health Organization
